# Floating-embedded stems reduce tibial stress shielding in total knee revision arthroplasty

**DOI:** 10.1186/s13018-025-05465-0

**Published:** 2025-01-16

**Authors:** Dominic Schaefer, Artur Barsumyan, Kambiz Roshanghias, Jan Adriaan Graw, Christian Soost, Wolfgang Richter, Jonas Knoche, Arne Ohrndorf, Rene Burchard

**Affiliations:** 1 Department of Engineering, School of Science, University of Siegen, Siegen, Germany; 2 University of Marburg, Marburg, Germany; 3 Department of Orthopaedics and Traumatology, University of Giessen and Marburg, Marburg, Germany; 4 Department of Orthopaedics and Trauma Surgery, Lahn-Dill-Kliniken, Dillenburg, Germany; 5 Department of Anesthesiology and Intensive Care Medicine, Ulm University Hospital, Ulm, Germany; 6 Department of Statistics, School of Economic Disciplines, University of Siegen, Siegen, Germany; 7 Department of Orthopaedics and Traumatology, University of Giessen and Marburg, Marburg Lahn-Dill-Kliniken, Rotebergstr. 2, 35683 Dillenburg, Germany

**Keywords:** Total knee arthroplasty, Revision surgery, Stress shielding, Aseptic loosening

## Abstract

**Background:**

Total knee arthroplasty (TKA) is one of the most common orthopaedic procedures and the number of patients which undergo TKA will continue to rise in the coming years. Consecutively, the number of necessary revision surgeries will increase. One of the main reasons for revision surgery is aseptic loosening because of a so-called stress-shielding effect. Typically, revision of a primary TKA is done from a bicondylar surface replacement to a stem-anchored prosthesis, which, due to higher stress-shielding, have a shorter survival time than non-stem-anchored systems. Similar to endoprosthetic treatment in pediatric tumor orthopedics, non-ingrown cementless stems can be used. The study aim was to investigate whether this concept can also be applied to reduce stress-shielding in the tibial metaphysis after revision TKA in adults.

**Methods:**

Six tibial biomechanical bone with stemmed tibial TKA components were implanted using surface cementing and a floating-embedded stem or classic full cementing. After implantation, axial force was applied in such a way that the same load was generated as during walking. Two high-resolution cameras and illumination spots were used to record changes on the bone surface circumferentially in three regions of interest and from three different views.

**Results:**

With regard to the fixation method, a significant difference could be demonstrated in the metaphyseal and in the middle region around the stem (*p* < 0.001). At the tip of the stems, the reduction of strain energy density showed a stress shielding reduction for the floating-stemmed models in two of three views (ventromedial *p* = 0.002, lateral *p* = 0.398, and dorsal: *p* = 0.027).

**Conclusions:**

In revision surgery after TKA, the use of floating-embedded, uncemented stems without bony ingrowth shows significant reduction of metaphyseal stress-shielding within the proximal tibia. This technique could be a viable alternative to prevent early aseptic loosening and should be examined in future in-vivo studies.

## Background

Total knee arthroplasty (TKA) is one of the most common orthopaedic procedures and the number of patients which have to undergo TKA will continue to rise in the coming years [1]. Consecutively, the number of necessary revision surgeries will increase. Considering an annual growth rate of 4.67%, about 115,000 revision surgeries associated with TKA are expected alone in the United States by 2040 [2]. One of the main reasons for TKA revision surgery is aseptic loosening of the TKA [3]. In addition to abrasion-induced inflammatory reactions, so-called stress-shielding within the framework of Wolff’s law leads to weakening of the implant bed [4]. The strain for normal remodeling of the proximal tibia should be within a physiological range of 50 to 1,500 µε [5]. Below 50 µε, stress shielding is likely to occur and bone resorption takes place. The risk of damage within the cancellous bone with microfractures increase above 1,500 µε, and the risk of collapse with pathological overloading increases above 3,000 µε [6, 7]. As a result of bone entering a reactive state if physiological strain range is left, this biomechanical conflict could cumulate in a deficiency of the bone-implant-interface. This circumstance often requires selection of an expanded and therefore more distal anchorage for the revision surgery using intramedullary stems.

Typically, revision of primarily TKA is done from a bicondylar surface replacement to a tibial and femoral stem-anchored prosthesis with different levels of coupling. The stem-anchored models offer a high primary loading capacity due to their placement in the unloaded bone farther from the joint. Nevertheless, stem-anchored prosthesis models are known to have a shorter survival time than non-stem-anchored system [8]. A main reason is the unloading of the metaphyseal bone in the context of stress-shielding. Au and colleagues were able to show that the soft metaphyseal bone of the proximal tibia is susceptible to this phenomenon [9]. Therefore, various research groups studied which anchoring principle could offer the longest survival. Maslaris and colleagues found advantages in implants provided with the shortest possible stems and a cemented fixation [10]. Others suggested longer stems without the use of bone cement [11]. In this scenario, cementless stems are implanted press-fit and appropriately coated for timely osteointegration [12, 13]. Ultimately, a superiority of one of these concepts could not be clearly demonstrated in a wide systematic review [14].

Known from the endoprosthetic treatment of children and adolescents in the context of tumor orthopedics, surgeons use non-ingrown cementless stems [15]. This way, impairment of the epiphyseal joint function or further growth of the leg due to the growing body is prevented.

The aim of the present study was to investigate whether the concept from pediatric orthopedics can also be applied to reduce stress-shielding in the tibial metaphysis after revision TKA in adults. We hypothesized that despite the use of stems, continuous force application occurs around the prosthesis-bone interface close to the joint. The resulting reduction in stress shielding might improve survival after revision arthroplasty as postulated by other study groups in finite element analyses [16, 17].

## Methods

### Study design and experimental setup

**Fig. 1:**
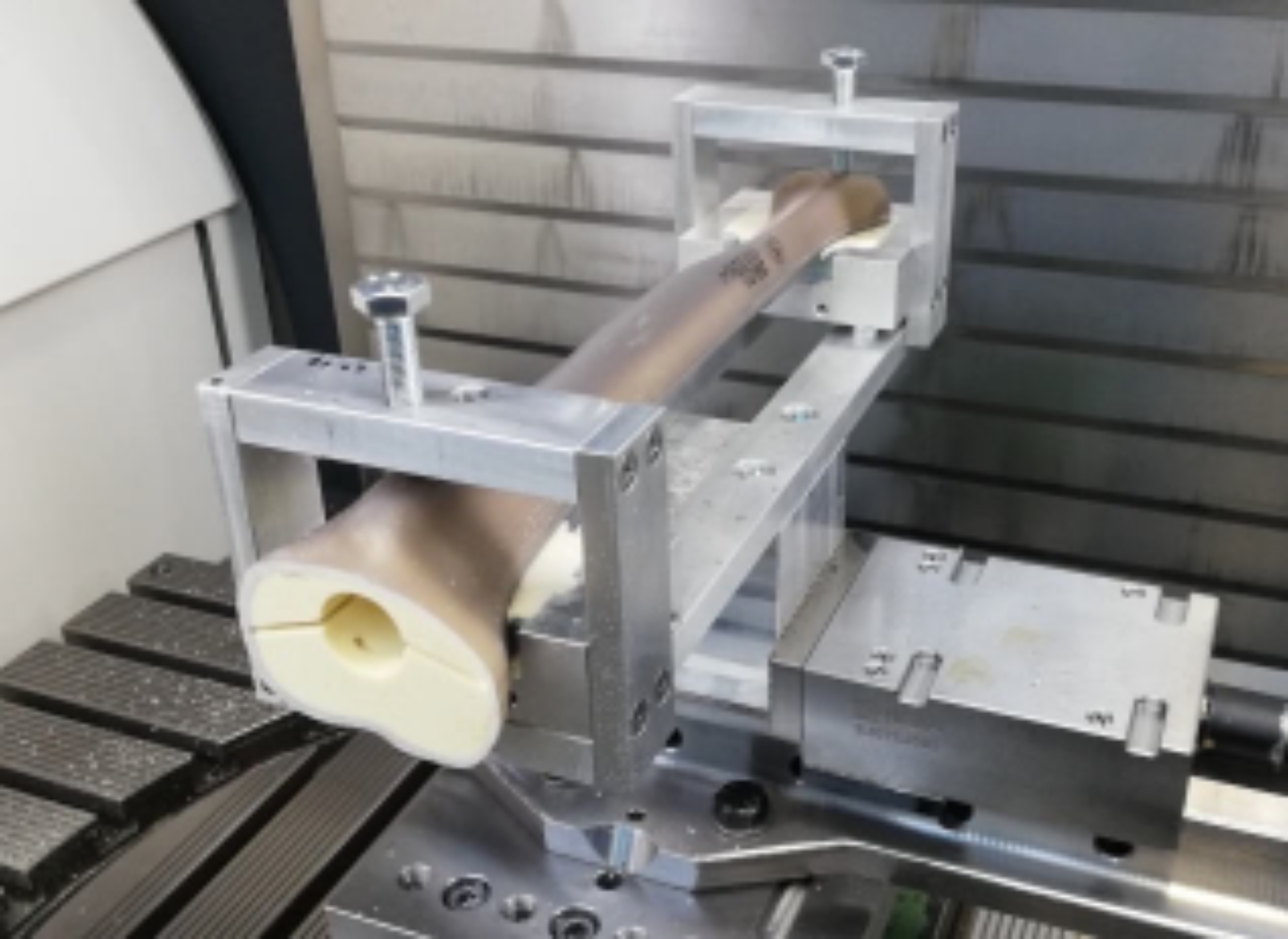
Fixed sawbone^®^ with a CNC prepared bed for implantation of the tibial component

A total of six tibial biomechanical bone models (Sawbone^®^, Sawbones, Vashon, USA) were used to investigate the different fixation methods. Prior to the implantation of the prostheses, CT (computer tomography) scans were taken to approve a consistent “preoperative” cortical thickness of all Sawbone^®^ samples. Based on these CT scans, three-dimensional planning was performed for implantation of a stemmed and constrained endoprosthesis at the tibial side (MUTARS GenuX^®^ modular, Implantcast GmbH, Buxtehude, Germany). The planning resulted in the combination of size 5 of the tibial component and a stem with the dimensions: 11 mm diameter and length of 125 mm (implavit^®^ (CoCrMo) for cemented or cementless use). The preparation of the implant bed was performed with a programmed CNC (computerized numerical control) milling machine in an exactly reproducible manner using a fixed mounting device (Fig. 1).

The prostheses were then implanted in three cases using surface cementing and a floating-embedded stem and in three cases using classic full cementing. Floating-embedded in this context means that the stem allows micromovements and is not connected to the bone by cementing or by ingrowth. According to the manufacturer’s manual the above mentioned implavit^®^ stem was used for both implantation techniques. Palacos^®^ R + G (Heraeus Medical GmbH, Wehrheim, Germany) was used as cement and implantations were performed by a board-certified senior knee surgeon. After implantation, CT scans documented the proper positioning of the implanted prostheses and also no difference in the bony conditions such as thickness or impaction of the cancellous bone.

### Method of investigation

**Fig. 2:**
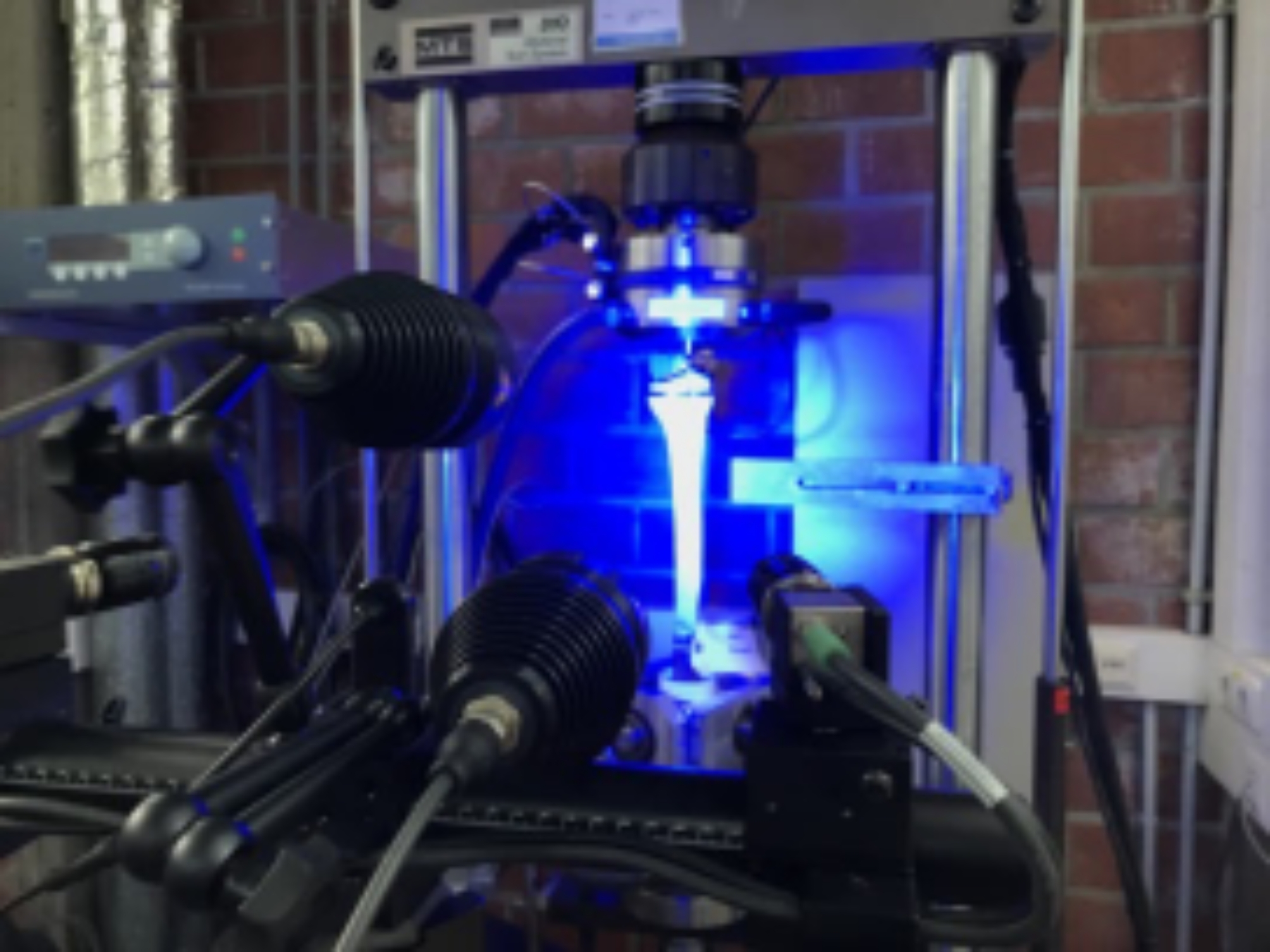
GOM aramis 12 M system with two cameras, a focusing laser and two spotlights, focused on coated Sawbone^®^ inside the testing machine

After implantation, the bone-prosthesis combinations were mounted in a testing machine MTS 810 Test Star 50 kN (MTS Systems GmbH, Berlin, Germany). The distal ends were clamped in a double-cardanic suspended device and proximal force application was established over the matching femoral counterpart of the tibial component (MUTARS GenuX^®^ modular, femoral component, size 5) (Fig. 2). A standard 12.5 mm polyethylene inlay was inserted, and the implant components were coupled. Forces were applied in such a way that the same load was generated as during walking. While maximum forces for an average normal weight of 75 kg are just under 2,000 N, for the typical overweight patients around 100 kg forces are slightly over 3,500 N [18]. Due to the high proportion of overweight patients in the population suffering from knee osteoarthritis, the higher forces were selected for this study.

Two high-resolution cameras and illumination spots (GOM Aramis 12 M, Carl Zeiss GOM Metrology GmbH, Oberkochen, Germany) were used to record changes on the bone surface circumferentially (Fig. 2). This system captures the surface of the Sawbone^®^, which was previously coated with a stochastic color pattern, using white matt lacquer as a base and black lacquer to create a speckled pattern. Based on the images, the software can determine the strains using digital image correlation. The results can be visually superimposed on the image of the sample within the program. This provides a local strain measurement and the entire strain field directly. The regions of interest (ROI) were defined according to Bourne and Finley as medial and lateral regions and supplemented by a third dorsal zone around the dorsal bone based on the three-dimensional data available from the GOM system [19].

### Statistical analysis

Data were analyzed using the open-source software R (R Core Team (2013). R: A language and environment for statistical computing. R Foundation for Statistical Computing, Vienna, Austria). All three observation planes (ventromedial, dorsal and lateral) are analyzed using a three-factor mixed-design ANOVA (Analysis of Variance). Two factors include repeated measures, and one factor varies between subjects. The two-factor repeated measures concern the different bone regions and the different loadings. The two fixation methods (cemented and cementless) represent the between-subject factors. Subsequent pairwise comparisons are corrected using the Bonferroni method. A P-value < 0.05 was considered statistically significant.

## Results

The indirect measurement of strain change in tibial direction (εy) was performed circumferentially. Stress between the two stem fixation methods (cemented and cementless, floating-embedded) differed in nearby all observation planes of ventromedial, dorsal and lateral. Only the area around the tip of the stem on the lateral view showed no difference. Accordingly, stress reduction as an indicator of stress shielding was grossly lower with the use of a floating-embedded, cementless stem.

### Axial strain of the tibia, optically measured by GOM aramis system

**Fig. 3:**
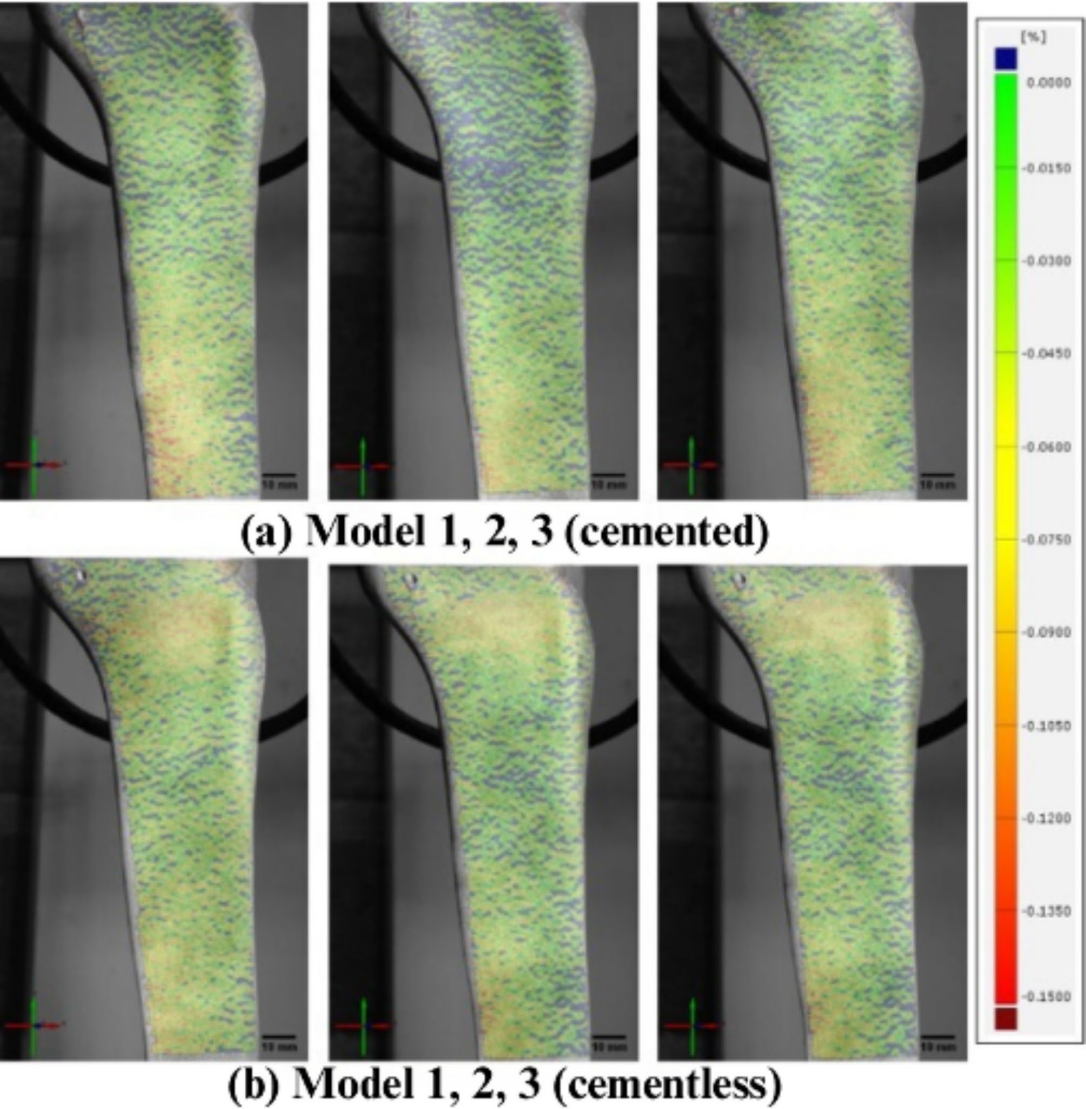
Overview of all samples from ventromedial side with displayed field of strains εy [%] (tibial axis direction) at 3,000 N

**Fig. 4:**
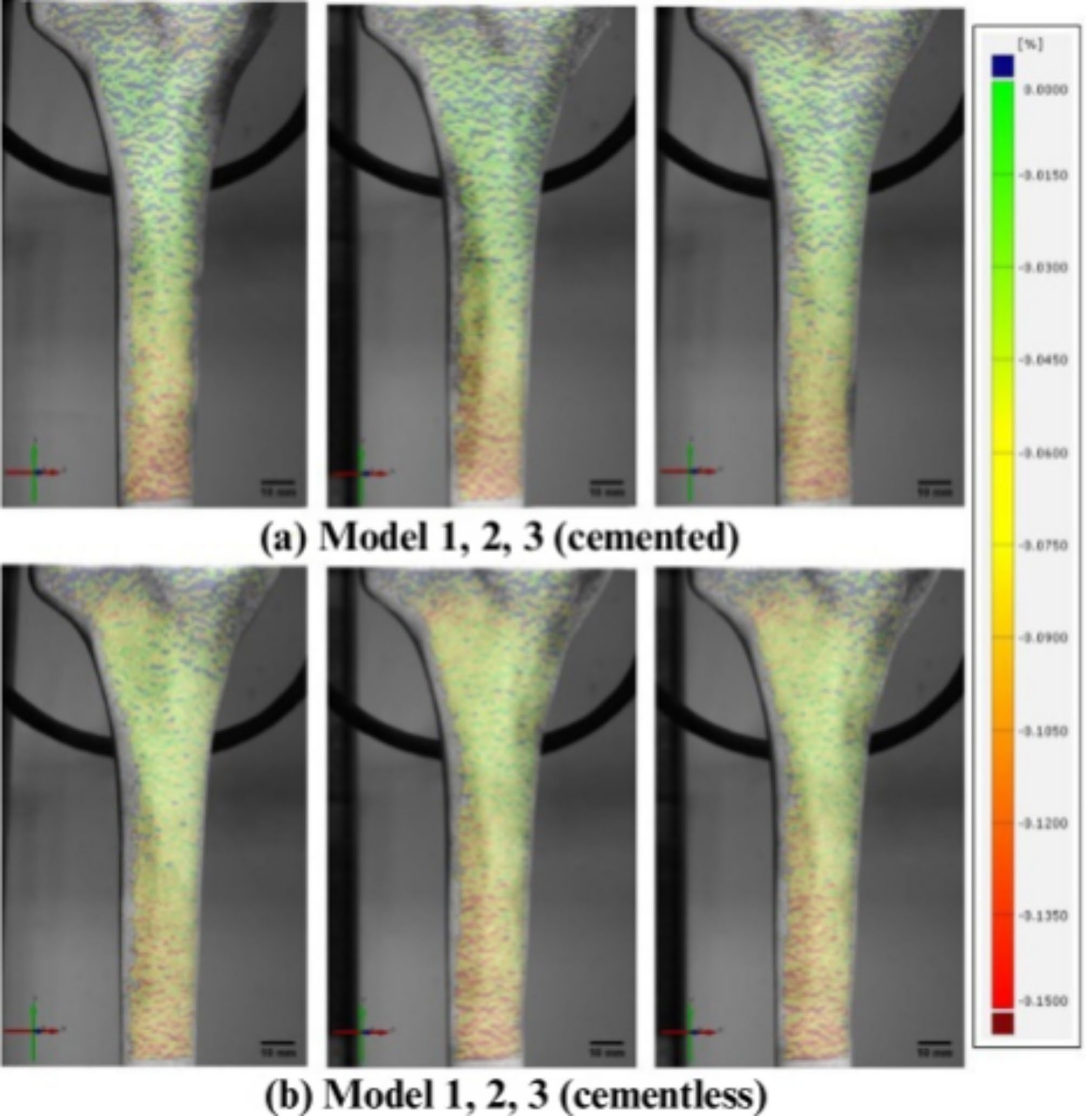
Overview of all samples from dorsal side with displayed field of strains εy [%] (tibial axis direction) at 3,000 N

**Fig. 5:**
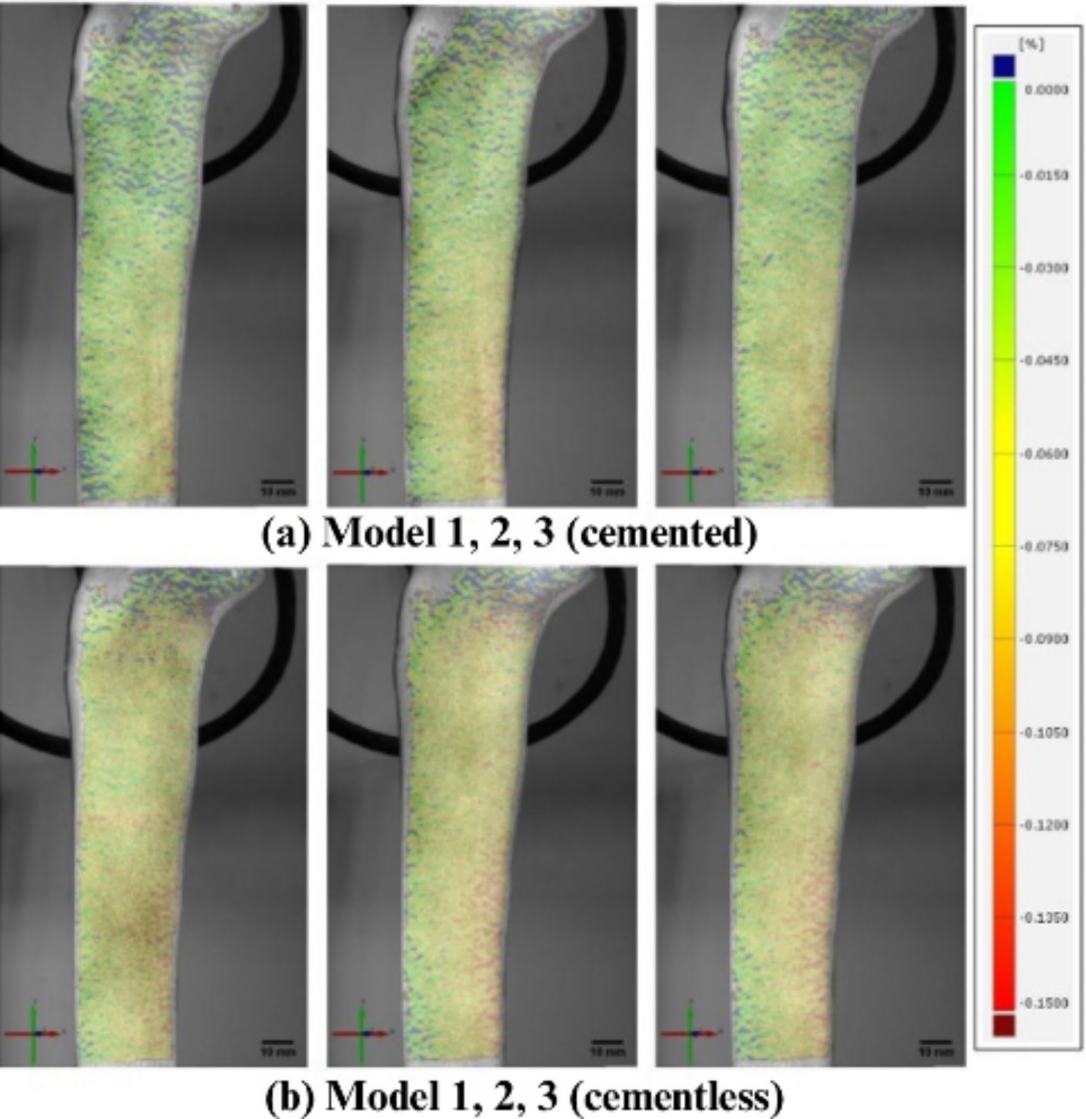
Overview of all samples from ventrolateral side with displayed field of strains εy [%] (tibial axis direction) at 3,000 N

On the ventromedial surface of the cemented models (Fig. 3a), the maximum strain (red) was visible in the distal-dorsal region, which corresponds to the stem end. The minimal strain values (green) occurred predominantly in the proximal area of the model. On the dorsal surface (Fig. 4a), a relatively high strain gradient was observed in the region of the stem end. The lateral surface (Fig. 5a) also showed the highest strain in this region.

On the ventromedial surface of the cementless, floating-embedded models (Fig. 3b), a field with maximum strain (red) was visible in the distal-dorsal region at the stem end. Less strain (green) was observed ventrally. In addition, another area with medium to maximum strain (yellow to red) was visible in the proximal metaphyseal region.

The measurements of the dorsal strain field showed a similar behavior as on the ventromedial side (Fig. 4b). The maximum strains (red) in the distal-dorsal area expanded dorsally. Proximally, above the lower maximum strain, lower strains were initially registered until they increased again in the metaphyseal region.

The lateral surface of the model with cementless, floating-embedded stems showed increased strains over the entire area, apart from the ventral area (Fig. 5b). The maximum strains were oriented dorsally along the entire diaphysis.

### Impact of stress on implants viewed from CT scan

**Fig. 6:**
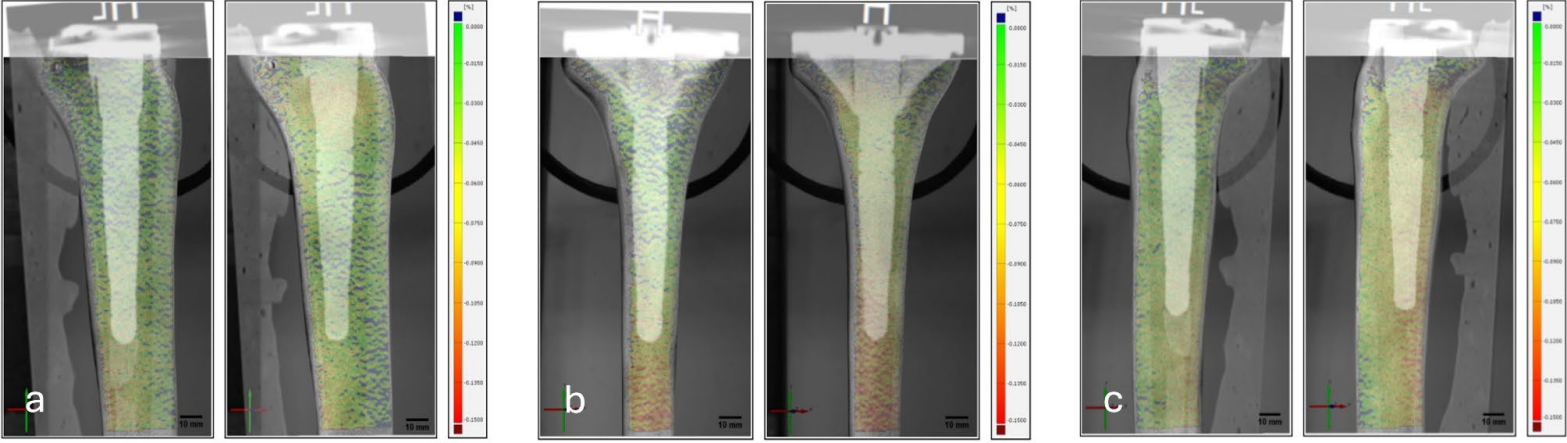
Displayed field of strains εy [%] by CT scans from ventromedial (**a**), dorsal (**b**) and ventrolateral (**c**)

Direct comparison between the cemented and the non-cemented fixation method according to implant’s position was illustrated using CT images. In the images of the cemented model (Fig. 6, left in each plane), the distal strain was increased, particularly at the end of the stem. In the non-cemented, floating-embedded model (Fig. 6, right in each plane), the strain was increased around the stem end but also in the metaphyseal area.

### Strain energy density changes in different periprosthetic regions of interest

**Fig. 7:**
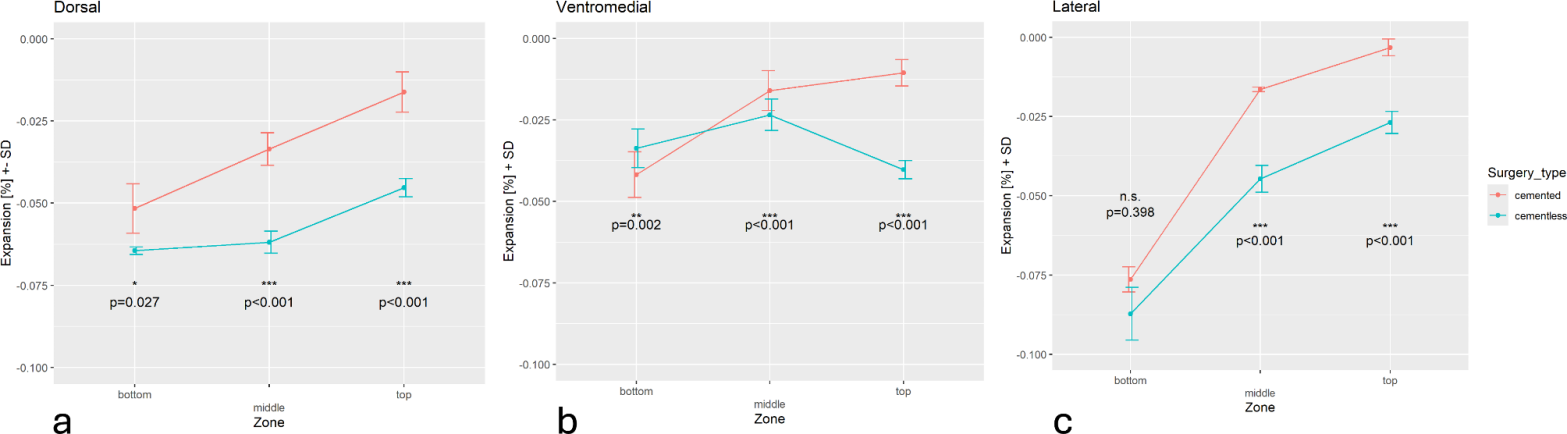
Differences of strain density according to the different fixation methods (red = cemented, green = cementless, floating-embedded) for the three defined periprosthetic regions of interest (top = metaphyseal region, middle = stem region, bottom = stem’s tip region) for the dorsal, ventromedial, and lateral measurements

To detect the influence of the different fixation methods on the regions of interest around the implanted prostheses, three major regions were defined by dividing the whole periprosthetic bone into three parts: metaphyseal region beneath the tibial plate, stem region, and stem tip region. After formation of the mean value of the mentioned three regions, all views (ventromedial, lateral, and dorsal) show a significant difference in the metaphyseal region with regard to the fixation method (*p* < 0.001). In the middle region around the stem complementary result were recognized with a highly significant reduction of stress shielding when a floating-embedded fixation was used (ventromedial, lateral, and dorsal view: *p* < 0.001). At the tip of the stems the reduction of strain energy density was not so prominent but also showed a stress shielding reduction for the uncemented models in two views (ventromedial *p* = 0.002, lateral *p* = 0.398, and dorsal: *p* = 0.027). Figure 7 illustrates the different effects of the two fixation models on strain density as a parameter of stress shielding.

## Discussion

In TKA loosening of the tibial component is identified as a major challenge [20]. In subsequent revision arthroplasty the focus is on preserving bone biology and stability as much as possible using intramedullary stems as a stabilizing anchor [21].

Since stress-shielding is known as a major reason for aseptic loosening, studies on this topic have produced conflicting and inconsistent results, with no distinct recommendations for procedural properties like stem fixation methods or implant dimensions. In their in-vitro studies, Bourne and colleagues demonstrated increased stress-shielding in the cortical bone contact zone of the proximal tibia with prostheses having an intramedullary stem [19]. Ritter and colleagues attributed tibial component loosening in cemented prostheses to inadequate preparation of the tibial resection surface due to insufficient jet lavage and lack of pressurization during cementing [22]. Other researchers suggested that factors influencing stress-shielding include the implant material, the design, and the surface characteristics of the prosthesis, as well as the bone condition at the time of implantation [23].

Cementless fixation of the tibial component in TKA was criticized for a long time [24]. Based on studies that linked complications such as stress shielding to cementless implantation, Lombardi and colleagues considered cemented implantation of the tibial component to be the gold standard for knee resurfacing [25]. Conversely, some researchers advocated for cementless fixation of total knee endoprostheses for superior longevity [13, 26]. Landon and colleagues promoted cementless fixation future-proof for increasingly younger and more active patients [27].

On the other side, in a meta-analysis including 34 studies and a mean follow-up period of 57.5 ± 26.4 months, no differences in revision rates, failure rates, aseptic loosening or periprosthetic infections between cemented and cementless fixation methods could be found [14]. Rozkydal and colleagues reported better results with cementless long stems in revision implants with poor bone quality [28]. Brooks and colleagues suggested that longer (cemented or cementless) stems are advantageous in cases with insufficient bone material, as they distribute the load evenly and protect the metaphyseal bone [29]. Completo and colleagues used finite element analyses to investigate if stem geometry impacts stress shielding more than material properties (cemented and cementless) [30]. Short stems caused less stress shielding in comparison to longer stems, while no significant differences were found between cobalt-chrome and titanium stems [30]. All dimensional variations showed high stress concentrations at the end of the stem. Bourne and Finlay’s cadaver study also indicated higher lateral strain, suggesting greater lateral compressive loading [19]. These findings are concordant with findings of Correa and colleagues, who demonstrated reduced tibial strain shielding using an extracortical plate system compared to cemented intramedullary fixation [31]. Another study group attributed varied stress shielding results to cementing techniques, cement volume, and penetration depth, which depended on the surgeon’s skills and preferences [32]. Furthermore, the role of micromovements is discussed controversially. On the one hand micromovements are known to cause prosthesis failure due to aseptic loosening [33]. On the other, potential reduction of stress shielding could occur by mechanical stimuli to the tibial plateau [17]. This complexity necessitates careful evaluation of micromovements to identify their impact on stress shielding during TKA.

The study presented here supports some of the previous scientific findings: in the cemented specimens, the maximum strains along the tibial axis were mainly concentrated in the distal area around the stem end. The strain distributions on the ventromedial, dorsal and lateral surfaces clearly show that the highest tensile and compressive strains are localized at the stem end. The strain decreased significantly proximally towards the metaphyseal area. In contrast, the cementless, floating-embedded models showed increased strains at the stem end and pronounced strain maxima in the metaphyseal region. These were particularly noticeable on the ventromedial and dorsal surfaces and showed high strain activity over a larger area along the tibial cortex. Overlaying the strain fields with radiological imaging data from computed tomography of the implanted components illustrated this difference. Cemented specimens showed high strains mainly concentrated immediately distal to the stem tip, while non-cemented, floating-embedded fixations showed high strains over larger proximal areas along the entire tibial cortex. Taken together, the results show that the observed strains in the non-cemented, floating-embedded group are associated with significant force transmission from the resection plane through the metaphyseal region.

### Limitations

Synthetic composite bones, such as Sawbones^®^, which are made from composite materials, are essential for the research and development of orthopedic devices. These replicas offer clear advantages over human bones as they are readily available, have low inter-sample variability and are easy to handle. They also have mechanical properties like those of normal, healthy bone. However, bone replicas do not allow realistic replication that does justice to the complexity of biomechanics and the interaction of biological systems, which can affect the validity of the experiments [34]. Furthermore, after surgical impaction of endoprostheses, bony conditions may be affected depending on the pressure condition and lead to deformation of the Sawbones’^®^ surface [35, 36]. To avoid this cofounding issue, we’ve performed pre- and postoperative CT scans that showed identical dimensions of the models used. However, the method used in this study is not capable of detecting changes in bone density caused by cancellous bone micro-impactions, as is the case with FE (finite elements) or DEXA (dual energy X-ray absorptiometry) studies [37, 38].

In the study presented, a simplified model of the real conditions was used, which only simulated a single axial load without considering shear forces or the influence of muscles and ligaments. In addition, to simplify the test parameters, a uniform layer thickness was assumed for all samples. The CT scans also suggest this, but it cannot be measured exactly.

Another limitation is the presence of noise in optical strain measurements, which limits the reliability of the strain field measurements. Although the noise peaks were distinctly below the maximum strain values, they reached measurable values. Noise in optical strain measurement, as in our case with the GOM system, can affect the reliability of the data [39]. This is also the reason why the evaluation with only 3 ROIs was selected in this study in order to minimize the effect of noise. Therefore, the application of filters, which were not available in this study, could be a solution for noise reduction in future series of experiments [40].

## Conclusions

In revision surgery after TKA, the use of floating-embedded, uncemented stems without bony ingrowth shows significant reduction of metaphyseal stress-shielding within the proximal tibia. This technique could be a viable alternative to prevent early aseptic loosening and should be examined in future in-vivo studies.

## Data Availability

No datasets were generated or analysed during the current study.

## Abbreviations

TKA: Total knee arthroplasty
CT: Computer tomography
CNC: Computerized numerical control
ANOVA: (Analysis of Variance)
ROI: (Region of Interest)
FE: (Finite elements)
DEXA: (Dual energy X-ray absorptiometry)
